# MicroRNA-550a Acts as a Pro-Metastatic Gene and Directly Targets Cytoplasmic Polyadenylation Element-Binding Protein 4 in Hepatocellular Carcinoma

**DOI:** 10.1371/journal.pone.0048958

**Published:** 2012-11-07

**Authors:** Qi Tian, Linhui Liang, Jie Ding, Ruopeng Zha, Haibing Shi, Qifeng Wang, Shenglin Huang, Weijie Guo, Chao Ge, Taoyang Chen, Jinjun Li, Xianghuo He

**Affiliations:** 1 State Key Laboratory of Oncogenes and Related Genes, Shanghai Cancer Institute, Renji Hospital, Shanghai Jiao Tong University School of Medicine, Shanghai, China; 2 Qi Dong Liver Cancer Institute, Qi Dong, Jiangsu, China; The University of Hong Kong, China

## Abstract

MicroRNAs (miRNAs) are a class of small, non-coding RNA molecules that are often found at chromosomal breakpoints and play a vital role in human cancer. Our previous study found that miR-550a, a frequently amplified miRNA on 7p14.3, was upregulated in hepatocellular carcinoma (HCC). However, the possible functions and molecular mechanisms of miR-550a in HCC remain unknown. In this study, gain-of-function and loss-of-function assays revealed that miR-550a markedly promoted HCC cell migration and invasion. In addition, we discovered that cytoplasmic polyadenylation element binding protein 4 (*CPEB4*) was a potential target of miR-550a in HCC. Further analyses showed that knockdown of *CPEB4* expression significantly facilitated HCC cell migration and invasion, which phenocopied the effects of miR-550a on HCC cells. Moreover, a decrease in *CPEB4* expression mediated miR-550a-induced liver cancer cell migration and invasion. Interestingly, *CPEB4* is frequently downregulated in HCC, and its expression levels correlate with the overall survival of HCC patients. Together, these results suggested that this newly identified miR-550a-*CPEB4* axis may be involved in HCC cell metastasis. Moreover, the expression levels of *CPEB4* could be used to predict outcomes in HCC patients. Our findings provide novel potential targets for HCC therapy and prognosis.

## Introduction

MicroRNAs (miRNAs) are small, single-stranded, non-coding RNAs that are highly conserved between species [1]. They are key post-transcriptional regulators of gene expression that act mainly via binding to the 3′ untranslated regions (3′ UTRs) of target mRNAs and thus participate in various biological processes [2], [3], [4], [5]. It has been predicted that miRNAs could regulate 60% of human genes, which makes miRNAs a powerful regulator in human physiology and pathology, including cancer [4], [6], [7], [8]. In the last decade, emerging evidence has indicated that miRNAs are differentially expressed and play critical roles in many cancers [6], [9].

Hepatocellular carcinoma (HCC) is one of the most prevalent cancers, particularly among East Asian and Southeast Asian populations [10]. Half of HCC cases and deaths worldwide are estimated to occur in China [11]. Despite its high lethality, the molecular mechanism underlying HCC remains largely unknown. As one hallmark of cancer, genomic instability leads to the over- or under-expression of genes and enables cancer cells to acquire multiple mutations, which leads to the initiation and development of cancer phenotypes [6], [12], [13], [14].

In the last decade, studies have revealed many chromosomal breakpoints related to genomic instability and identified numerous oncogenes/tumor suppressors that are involved in cancer pathogenesis. However, the non-coding RNAs located at chromosome breakpoints are largely unknown. Our laboratory has extensively analyzed the miRNAs in common recurrent chromosomal aberration regions and identified some miRNAs that were aberrantly expressed in HCC [12]. Among the miRNAs associated with the chromosomal breakpoints, miR-151 and miR-30d were found to be critical regulators of HCC invasion and metastasis. However, the roles of the remaining miRNAs identified are still unexplored in HCC. Therefore, we performed a preliminary functional screen of the remaining miRNAs and found that miR-550a could regulate HCC cell motility. miR-550a is located in 7p14.3, which is frequently amplified in many cancers, such as gastric carcinomas [15], malignant peripheral nerve sheath tumors (MPNSTs) [16], [17], malignant mesotheliomas (MMs) [18], nasopharyngeal carcinoma (NPC) [19], esophageal squamous cell carcinoma (ESCC) [20], seminomas [21] and HCC [22]. Gene gains have also been reported at this site. For example, a gain of TCRG (TCR gene loci) was observed in T-cell lymphoma [23]. These findings suggested that genes located in this site may play a role in the development of cancers and hinted at a role for miR-550a in HCC.

**Figure 1:**
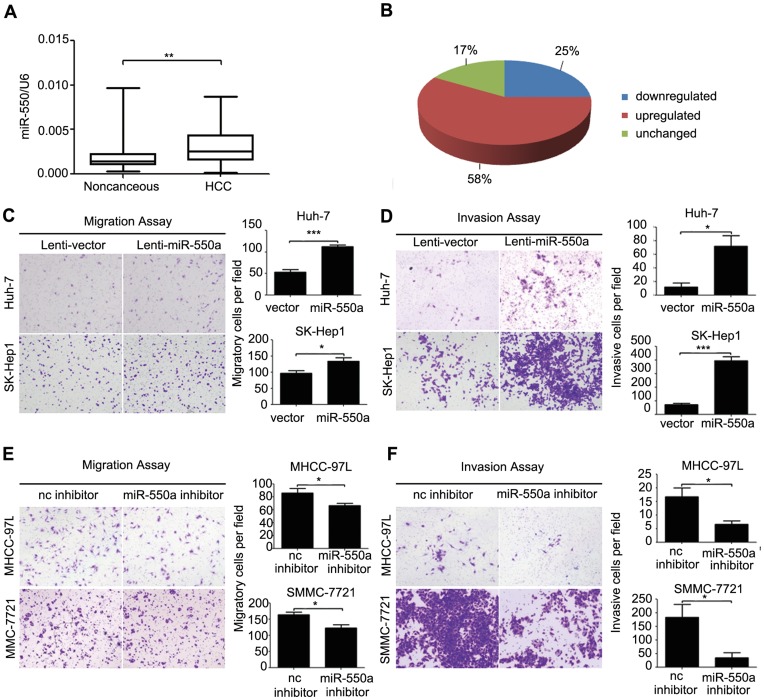
miR-550a is often upregulated in HCC and promotes HCC cell migration and invasion. (**A, B**) The relative expression of mature miR-550a in 48 pairs of HCC tissues and their corresponding noncancerous liver tissues were measured using TaqMan real-time PCR and normalized to U6 snRNA. (**C, D**) Transwell migration and invasion assays of Huh-7 and SK-Hep1 cells stably expressing miR-550a or mock control. (**E, F**) Transwell migration and invasion assays of MHCC-97L and SMMC-7721 cells transfected with a miR-550a inhibitor or negative control. Representative images are shown on the left, and quantification is shown on the right. The results are representative of at least three independent experiments, and the values shown are the mean ± SD.

In this study, we verified that the expression of miR-550a is significantly upregulated in HCC tissues compared with noncancerous tissues. Ectopically expressed miR-550a could promote the migration and invasion of liver cancer cells. Furthermore, we found that *CPEB4* is a direct and functional target of miR-550a. The expression of *CPEB4* is closely correlated with therapeutic outcomes in HCC patients.

## Materials and Methods

### Human Liver Tumor Samples/Ethics Statement

HCC, the matched noncancerous liver tissues (3 cm from the tumour) and the cirrhosis liver tissues were obtained from the surgical specimen archives of the Qidong Liver Cancer Institute, Jiangsu Province, China. Ten normal liver tissue samples were obtained from people who died from accidents. Participants that these samples were obtained from provided their written informed consent to participate in the study, and the Ethical Review Committee of the WHO Collaborating Center for Research in Human Production authorized by the Shanghai Municipal Government approved this study as well as the consent procedure.

**Figure 2:**
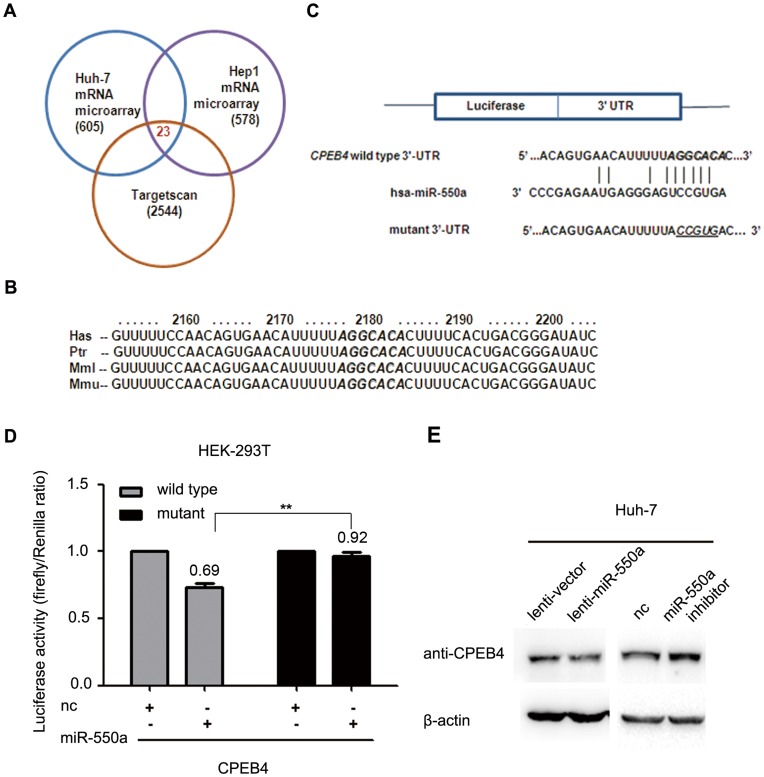
miR-550a downregulates *CPEB4* expression by directly targeting its 3′UTR. (**A**) Scheme for the identification of candidate genes combining microarray assays and a target prediction algorithm. (**B**) The putative binding sequences for miR-550a within the human (Has), chimpanzee (Ptr), rhesus (MMl) and mouse (Mmu) *CPEB4* 3′UTR. Seed sequences are indicated as emphasized with shadow. (**C**) The sequences of the putative miR-550a-binding site in the wild-type (emphasized with shadow) and mutant (underlined) *CPEB4* 3′UTR. (**D**) The relative luciferase activity of luciferase reporters with wild-type or mutant *CPEB4* 3′UTRs was determined in HEK 293T cells, which were co-transfected with the miR-550a mimic or negative control (nc). Renilla luciferase activity was analyzed as an internal control. There is statistical significance between the wild-type group and the mutant group with miR-550a transfection. Representative experiments are shown with the mean ± SD. (**E**) The protein levels of *CPEB4* were determined by western blot assays in Huh-7 cells infected with miR-550a or mock vector and Huh-7 cells transfected with the miR-550a inhibitor or negative control (nc).

### Cell Culture

HEK 293T, HepG2, Huh-7, SK-Hep-1, SMMC-7721, MHCC-LM3 and MHCC-97L cells were cultured in Dulbecco’s modified Eagle’s medium (DMEM) with 10% fetal bovine serum (FBS) and antibiotics. SNU-449 cells were cultured in RPMI-1640 medium supplemented with 10% FBS and antibiotics.

### RNA Extraction and Quantitative Real-time PCR

Total RNA was extracted with TRIzol reagent (Invitrogen). cDNA was synthesized with the PrimeScript RT reagent Kit (TaKaRa). Real-time PCR was performed with SYBR Premix Ex Taq (TaKaRa). Mature miRNAs were quantified with specific primers and probes using TaqMan microRNA Assays (Applied Biosystems). The primers used are listed in Table S1.

### Vector Constructs

In the miR-550a lentivirus expression vector pWPXL-miR-550a, the primary miRNA sequence amplified from normal genomic DNA replaced the green fluorescent protein fragment of the pWPXL mock vector. In the luciferase reporter vector, the wild-type or mutant 3′UTR of *CPEB4* was cloned downstream of the stop codon in the luciferase gene. Other potential target genes were cloned in a similar manner. The sequences of the primary miRNA and wild-type and mutant 3′UTR were confirmed by sequencing. The primers and the detail of miR-550a vector as well as 3′UTRs are separately listed in Table S2 and S3.

**Figure 3:**
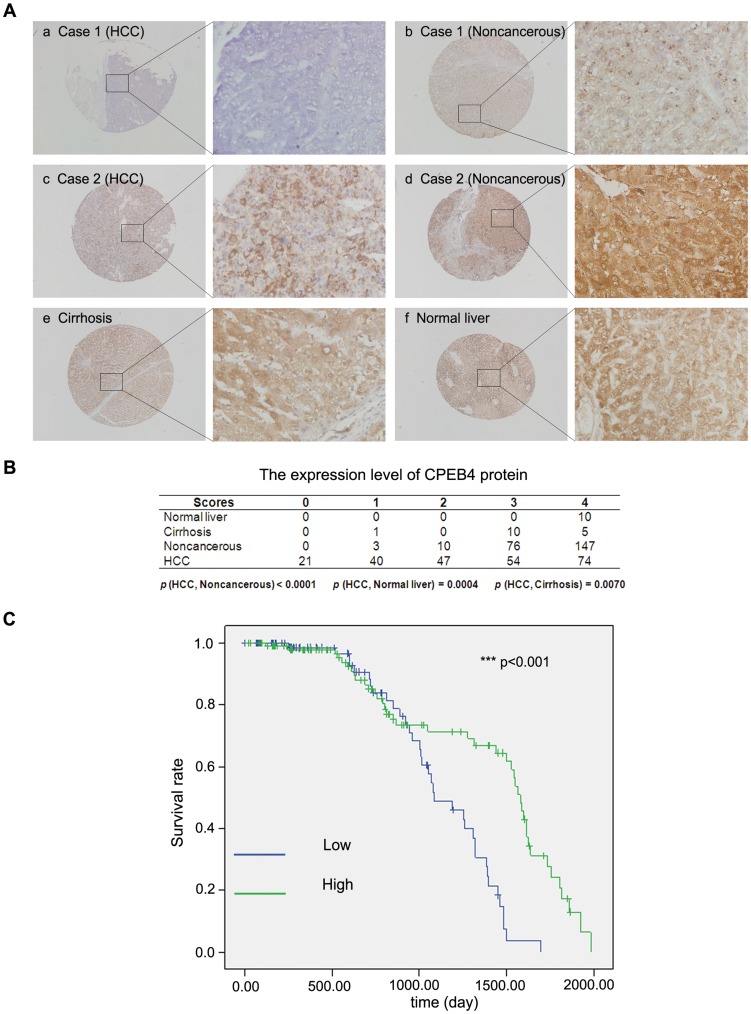
*CPEB4* is often reduced in HCC and correlated with the overall survival of HCC patients. (**A, B**) Immunohistochemical staining of *CPEB4* in HCC tissues, paired adjacent noncancerous tissues, cirrhosis tissues and normal liver tissues. The staining was scored and represented as follows: a, score 0; b, score 3; c, score 2; d, score 4; e, score 3; f, score 4. The statistical results are shown in tables. Normal liver tissues, n = 10; cirrhosis liver tissues, n = 16; noncancerous tissues, n = 236; HCC, n = 236. (**C**) The survival curves of HCC patients are shown for low (score = 0−2) or high (score = 3−4) *CPEB4* expression level (*p*<0.01). The statistical analyses of cases in groups according to the scoring were performed with the χ^2^ test and Kaplan-Meier plots.

### Lentivirus Production and Transduction

Virus particles were harvested from HEK 293T cells 48 h after pWPXL-miR-550a transfection with the envelope plasmid pMDG2 and the packaging plasmid psPAX2 using Lipofectamine 2000. Huh-7 and SK-HEP-1 cells were infected with recombinant lentivirus-transducing units and 6 µg/mL polybrene.

### Oligonucleotide Transfection

The miRNA mimics and small interfering RNAs (siRNA) targeting *CPEB4* were designed and synthesized by GenePharma. The miR-550a inhibitor was synthesized by RiboBio. Cells were transfected with mimic or inhibitor using Lipofectamine 2000, while siRNA transfection was performed with Lipofectamine RNAi MAX reagents according to the manufacturer’s instructions (Invitrogen). Commonly, 48 hours after transfection, cells were used in experiments. The sequences of siRNAs used are shown in Table S4.

**Figure 4:**
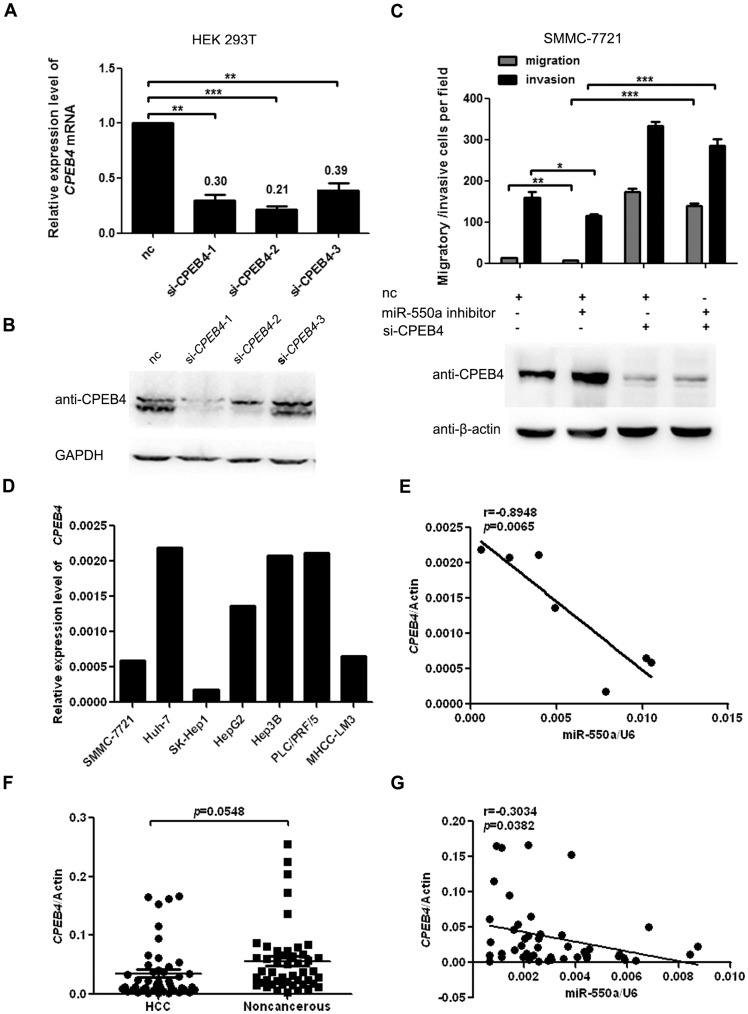
*CPEB4* suppresses miR-550a-induced migration&invasion and its expression is inversely correlated with miR-550a in HCC. (**A, B**) Real-time PCR and western blot of *CPEB4* expression in HEK 293T cells transfected with siRNAs targeting *CPEB4* or a negative control (nc). In (A), there is statistical significance between the nc group and either the si-RNA group. And the data are shown as the mean ± SD. (**C**) Transwell migration and invasion assays of SMMC-7721 cells were performed after transfection with a miR-550a inhibitor, *CPEB4* siRNA or a negative control (nc). The protein level of *CPEB4* was detected through western blot assays. There is statistical significance between group 1 and group 2 as well as between group 2 and group 4. The results are presented as the mean ± SD. (**D**) The relative expression of *CPEB4* at the mRNA level was analyzed through real-time PCR, normalized to β-actin. (**E**) The correlation between *CPEB4* expression and mature miR-550a in various liver cancer cell lines was analyzed by linear regression. (**F**) The mRNA level of *CPEB4* was determined in 48 pairs of HCC tissues and matched noncancerous liver tissues by real-time PCR. (**G**) The correlation between *CPEB4* expression and mature miR-550a was analyzed in the same HCC samples by linear regression. The expression data were normalized to β-actin and U6 snRNA, respectively.

### Cell Proliferation Assays

Cell proliferation was assessed by the Cell Counting Kit-8 (CCK-8) assay kit (Dojindo Corp.). Approximately 10^3^ cells were seeded in each well of a 96-well plate, and 10 µL CCK-8 was added to 90 µL culture medium. After incubation at 37°C for 2 h, the absorbance was detected at 450 nm and the OD450 value is correlated with the number of live cells.

### Migration and Invasion Assays

A 24-well plate containing 8 µm-pore size chamber inserts (BD Biosciences) was used to evaluate the migration and invasion of tumor cells. For the invasion assay, the membrane was coated with Matrigel to form a matrix barrier, and 10^5^ cells were placed in the upper chamber. For the migration assay, only 5×10^4^ cells were seeded in the upper chamber. In each lower chamber, 800 µL of Dulbecco’s modified Eagle’s medium (DMEM) with 10% fetal bovine serum (FBS) was added. After several hours of incubation at 37°C, cells that had migrated through the pore were fixed and stained with a mixture of 20% methanol and 0.1% crystal violet for 0.5 h. Then, the cells were photographed and counted under an IX71 inverted microscope.

### Luciferase Reporter Assay

HEK 293T cells were cultured in 96-well plates and transfected with 50 ng pluc-3′UTR, 10 ng Renilla and 5 pmol miR-550a mimic or negative control. After 48 h of incubation, luciferase activity was detected with the dual-luciferase reporter assay system (Promega).

### Western Blot Assays

Cell lysates were separated with 10% SDS-PAGE and transferred to a nitrocellulose membrane. The membrane was incubated with rabbit anti-*CPEB4* polyclonal antibody (Abcam, Sigma), mouse anti-β-actin (Sigma) or mouse anti-GAPDH monoclonal antibody (Kangchen). The proteins were visualized using enhanced chemiluminescence reagents (Thermo Scientific).

### Immunohistochemical Staining (IHC)

All of the samples were processed to analyze the expression of the *CPEB4* protein in HCC tissues, matched noncancerous tissues, cirrhotic tissues and normal liver tissues. Paraffin-embedded tissues were cut into 5 µm thick sections and analyzed with IHC using a rabbit antibody against *CPEB4* (Sigma). Scoring was based on the proportion of positively stained cells: <5% was scored as 0; 5–24% was scored as 1; 25–49% was scored as 2; 50–74% was scored as 3; and more than 74% was scored as 4.

### Statistical Analysis

The data are shown as the mean ± standard deviation (SD). Statistical analyses were performed with a two-tailed Student *t*-test unless otherwise specified. Differences were considered statistically significant at *p*<0.05. Asterisks were used to represent statistical significance of *p* values in some figures, e.g. *p≤0.05, **p≤0.01, ***p≤0.001.

## Results

### miR-550a is Frequently Upregulated in HCC and Accelerates HCC Cell Migration and Invasion

To validate the expression of miR-550a in HCC, we detected mature miR-550a in 48 pairs of HCC and matched noncancerous liver tissues by real-time PCR. The results indicated that miR-550a expression was upregulated in 58% of HCC tissues compared with the noncancerous liver tissues (Figure 1A, B). In addition, the endogenous expression of miR-550a in various liver cancer cell lines was evaluated. miR-550a expression was relatively low in Huh-7 cells, whereas SMMC-7721 and MHCC-97L cells had a relatively high basal level of miR-550a (Figure S1).

To examine the biological function of miR-550a in HCC, a miR-550a mimic was transfected into two HCC cell lines to determine whether it could affect cell proliferation and motility. CCK-8 assays suggested that miR-550a did not influence HCC cell growth (Figure S2A, B), and transwell assays with or without Matrigel indicated that miR-550a markedly induced migration and invasion in HCC cell lines (Figure S3A, B). Next, a lentivirus vector expressing miR-550a was constructed and used to infect Huh-7 and SK-Hep1 cells to establish stable cell lines because these cell lines exhibited relatively low endogenous miR-550a levels. The expression of miR-550a in the two stable cell lines was measured by real-time PCR (Figure S4). In transwell assays, Huh-7 cells overexpressing miR-550a showed enhanced migratory and invasive abilities compared with vector overexpressing cells (Figure 1C, D). Similar results were obtained in SK-Hep-1 cells (Figure 1C, D). To further confirm these findings, a miR-550a inhibitor was transfected into SMMC-7721 and MHCC-97L cells to suppress endogenous miR-550a. The transwell assays showed that the miR-550a inhibitor could significantly inhibit the migration and invasion of SMMC-7721 and MHCC-97L cells (Figure 1E, F). Taken together, these data demonstrated that miR-550a promoted HCC cell migration and invasion.

### miR-550a Downregulates *CPEB4* Expression by Directly Targeting its 3′ UTR

To clarify the molecular mechanism responsible for the effect of miR-550a on HCC cell migration and invasion, mRNA microarray assays were performed to identify the genes that were suppressed by miR-550a in both Huh-7 and SK-Hep1 cells transfected with the miR-550a mimic. At the same time, potential targets were predicted using the target-predicting algorithm TargetScan. By integrating the results of these two strategies, 23 genes were found to be possible targets of miR-550a (Figure 2A, Table S5). Subsequently, real-time PCR, dual-luciferase reporter analyses and western blot assays were carried out to verify whether any of these genes was actually regulated by miR-550a (Figure S5A, B and C). After preliminary screening, *CPEB4* was identified as a potential target of miR-550a in HCC which was downregulated by miR-550a at the mRNA and protein levels.

Next, analysis of the *CPEB4* 3′UTR sequence by TargetScan indicated only one possible binding site for miR-550a, which was highly conserved among humans, chimpanzees, rhesus and mice, *et al*. (Figure 2B, C). To further determine whether *CPEB4* is regulated by miR-550a through directly binding to its 3′UTR, a luciferase reporter vector containing the wild-type or mutant 3′UTR of *CPEB4* was constructed. After co-transfection with a miR-550a mimic, the luciferase activity was significantly reduced in the group that was co-transfected with the vector containing wild-type *CPEB4* 3′ UTR, while the luciferase activity was not reduced in the mutant 3′UTR group (Figure 2D). This result suggests that miR-550a binds to the 3′ UTR of *CPEB4* via the predicted binding site.

In addition, western blot analyses showed that high miR-550a expression could lead to a decreased *CPEB4* protein level in Huh-7 cells. In contrast, the miR-550a inhibitor increased the *CPEB4* protein level (Figure 2E). Collectively, these results indicated that miR-550a could negatively regulate *CPEB4* expression by directly binding to its 3′UTR.

### The Expression of *CPEB4* is Often Reduced in HCC, and its Protein Levels is Correlated with Survival Time in HCC Patients

Previous reports indicated that *CPEB4* plays a role in regulating translation, meiotic division and mitosis [24], [25], [26]. *CPEB4* was found to be upregulated in pancreatic ductal cancer and neuroblastoma [27]. However, the expression of *CPEB4* in HCC remains unknown. Therefore, we evaluated the expression of *CPEB4* in 236 HCC samples. The protein levels of *CPEB4* were analyzed with IHC staining. *CPEB4* expression was high (scored 3 or 4) in all 10 normal liver samples and in most of the cirrhosis samples and noncancerous cases. In contrast, *CPEB4* staining was relatively weak in almost half of the HCC samples (Figure 3A, B). Notably, the *CPEB4* protein levels showed a close correlation with the overall survival time of HCC patients (Figure 3C). There was no correlation between *CPEB4* levels and other clinicopathological factors such as age, sex and stage, et al. Taken together, these data suggest that *CPEB4* protein expression was frequently downregulated in HCC, and its expression was correlated with HCC prognosis.

### miR-550a Acts by Repressing *CPEB4* Expression in HCC

To clarify the effects of *CPEB4* in HCC cells, siRNAs against *CPEB4* were designed and transfected into HEK 293T cells. The mRNA and protein levels of *CPEB4* were markedly decreased, particularly in the si-*CPEB4*-1 group which was used in further experiments (Figure 4A, B). Next, SMMC-7721 cells were transfected with siRNAs and subjected to transwell assays. The results indicated that SMMC-7721 cells transfected with siRNA against *CPEB4* exhibited enhanced migration and invasion potential (Figure 4C). These results mimicked the phenotype induced by miR-550a overexpression and further suggested that *CPEB4* may be a functional target of miR-550 in HCC.

We next attempted to determine whether *CPEB4* was involved in miR-550a-induced HCC cell migration and invasion. The miR-550a inhibitor and siRNAs targeting *CPEB4* were co-transfected into SMMC-7721 cells in which miR-550a expression was relatively high. The subsequent transwell assays demonstrated that *CPEB4* knockdown partly neutralized the suppressive effects of the miR-550a inhibitor on HCC cell migration and invasion (Figure 4C). These data provided further evidence that *CPEB4* could inhibit miR-550a-induced HCC cell migration and invasion, suggesting that *CPEB4* is a direct and functional target of miR-550a in HCC.

If miR-550a actually regulates the expression of *CPEB4* in HCC, then the expression of these two factors should be inversely correlated in HCC. Therefore, we evaluated the expression of *CPEB4* mRNA in various liver cancer cell lines (Figure 4D). The results showed that the mRNA level of *CPEB4* was inversely correlated with the expression of miR-550a in these cell lines (Figure 4E). To extend our analysis to clinical cases, we assessed the mRNA level of *CPEB4* in the previous 48 cases of HCC and the adjacent noncancerous liver tissues. *CPEB4* mRNA was downregulated in HCC tissues compared with their respective noncancerous liver tissues (Figure 4F), consistent with the observed *CPEB4* protein levels. Moreover, the downregulation of *CPEB4* was correlated with the upregulation of miR-550a in these HCC samples (Figure 4G). These data suggest that *CPEB4* mRNA expression is negatively correlated with miR-550a expression in HCC.

## Discussion

In this study, we found that miR-550a is frequently upregulated in HCC and facilitates HCC cell migration and invasion. The direct, functional miR-550a target gene *CPEB4* is commonly suppressed in HCC, and its expression is correlated with HCC patient outcome.

According to miRBase, hsa-miR-550 is located in chromosomal region 7p14, and two members of the hsa-miR-550 family have been identified: hsa-miR-550a and hsa-miR-550b. In a previous study from our group, miR-550a was identified in a screen and referred to as miR-550-2 [12]. We found that the miR-550a DNA copy number was distinctly amplified in HCC, and its expression was evidently upregulated. In this study, we verified the upregulation of miR-550a in an independent cohort of HCC samples, and the results were consistent with those of our previous report. Recent studies have demonstrated that miR-550 is differentially expressed in gastritis and gastric extranodal marginal zone lymphoma and may have a role in the transition from gastritis to monoclonal B-cell lymphoma [28]. In addition, miR-550 is differentially expressed in childhood acute lymphoblastic leukemia [29]. Together with our results, these findings indicated that the deregulation of miR-550a could be common to several cancers and may have a functional role. Additionally, miR-550 has been found to be upregulated in prolactinomas following bromocriptine treatment [30]. Furthermore, miR-550 is among the eight microRNAs that predict sensitivity to prednisone in childhood acute lymphoblastic leukemia [29]. These results suggest that miR-550 may act as an indicator of treatment responses in different cancers and is worthy of further investigation. To the best of our knowledge, although miR-550 was found to be deregulated in several tumors, there is little data about the function of miR-550a. In this study, for the first time, we found that miR-550a could promote the migration and invasion of HCC cells. Meanwhile, miR-550a expression was associated with the vascular invasion of HCC, which may be due to the invasion-promoting function of miR-550a in HCC.

This mechanistic insight into the effect of miR-550a on cell migration and invasion suggested that the target gene *CPEB4* mediated the function of miR-550a in HCC. *CPEB4* belongs to the cytoplasmic polyadenylation element-binding protein family, the members of which mainly regulate translation by controlling the polyadenylation of target genes. The *CPEB* family contains two subfamilies, *CPEB1* and *CPEB2*. Although the biological function of *CPEB1* has been studied extensively, the function of *CPEB4*, a member of the *CPEB2* subfamily, remains largely unexplored. It has been reported that *CPEB4* acts as a cell survival protein in neurons [31] and regulates meiotic cells [25]. In pancreatic ductal cancer and neuroblastoma, the expression of *CPEB4* is upregulated, driving the growth and invasion of cancer cells [27]. In this study, we found that the mRNA and protein levels of *CPEB4* were often downregulated in HCC. The tissue-specific feature and some other factors which are unexplored may attribute to this phenomenon in different kind of tumor tissues. Furthermore, we found that *CPEB4* siRNA could promote the migration and invasion of HCC cells. Our results contradict those of a previous report. We assume that this contradiction might be due to the different downstream targets regulated by *CPEB4* in different cells because *CPEB4* can control the translation of many genes by binding to the CPE sequence in their 3′ UTR [32]. Intriguingly, relatively high levels of *CPEB4* predicted a better outcome in HCC patients. These results indicated that *CPEB4* could act as a prognostic factor in HCC. We also found that miR-550a and *CPEB4* expression were inversely associated in HCC samples, which suggested that the downregulation of *CPEB4* in HCC may be at least partially due to the upregulation of miR-550a. The regulation of *CPEB4* by microRNAs has also been reported by Morgan et al. [32]. They found that members of the *CPEB2* subfamily could be co-regulated by microRNAs through a conserved sequence in their 3′ UTR. Together with our results, these findings demonstrate that microRNA regulation is a common phenomenon in *CPEB4* regulation.

In summary, our findings show that increased miR-550a expression due to DNA amplification increased the migratory and invasive abilities of HCC cells. Knockdown of the miR-550a target gene *CPEB4* enhanced the migration and invasion of HCC cells. Importantly, *CPEB4* expression is correlated with HCC patient outcome, and miR-550a/*CPEB4* may represent a promising prognostic and therapeutic target in HCC.

## Supporting Information

Figure S1 **The relative expression of miR-550a in various liver cancer cells.** The relative expression level of mature miR-550a was detected by TaqMan real-time PCR. The data were normalized to U6 snRNA.
(TIF)

Figure S2 **miR-550a has no significant effects on HCC cell growth in vitro. (**A, B**)** CCK-8 assays of Huh-7 and HepG2 cells were performed every other day after transfection with a miR-550a mimic or negative control (nc). The data are presented as the mean ± S.E.M.
(TIF)

Figure S3 **A miR-550a mimic facilitates HCC cell migration and invasion in vitro. (**A) Transwell migration assays of Huh-7 and SMMC-7721 cells transfected with the miR-550a mimic or negative control (nc). (B) Transwell invasion assays of SMMC-7721 and SNU-449 cells transfected with the miR-550a mimic or nc. The values shown indicate the mean ± S.E.M.
(TIF)

Figure S4 **The expression of miR-550a in stable cell lines.** The relative expression level of mature miR-550a was determined in Huh-7 and SK-Hep1 cells infected with pWPXL-miR-550a or control lentivirus. U6 snRNA was used as an internal control.
(TIF)

Figure S5 **The identification of potential miR-550a target genes.** (A) The mRNA expression levels of the predicted genes in Huh-7 and SK-Hep1 cells expressing miR-550a or vector were evaluated by real-time PCR. (B) Dual-luciferase activity assays were used to determine the binding potential between miR-550a and the 3′UTR of these candidate genes. Renilla luciferase activity was detected as an internal control. (C) Western blot assays of the TRAK2 and *CPEB4* protein levels in Huh-7, SMMC-7721 and HEK 293T cells.
(TIF)

Table S1 **The primer sequences for real-time PCR.**
(DOC)

Table S2 **The nested PCR primer sequences for miR-550a or 3'UTR of potential target genes.**
(DOC)

Table S3 **The information of miR-550a and its 3′UTR vectors.**
(DOC)

Table S4 **The sequences of siRNAs against** ***CPEB4*** **.**
(DOC)

Table S5 **The possible target genes for miR-550a in HCC cells.**
(DOC)
