# Supplementary figures and images for: MicroRNA-550a Acts as a Pro-Metastatic Gene and Directly Targets Cytoplasmic Polyadenylation Element-Binding Protein 4 in Hepatocellular Carcinoma
Source: PLoS One. 2012 Nov 7;7(11):e48958. doi: 10.1371/journal.pone.0048958 (PMC3492136; doi:10.1371/journal.pone.0048958)

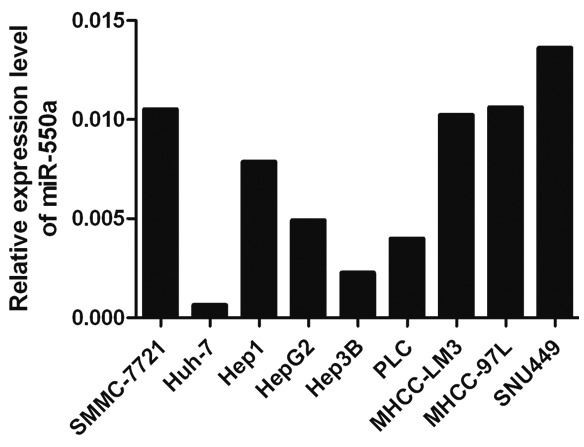

Supplement: Figure S1 — The relative expression of miR-550a in various liver cancer cells. The relative expression level of mature miR-550a was detected by TaqMan real-time PCR. The data were normalized to U6 snRNA. (TIF) [file pone.0048958.s001.tif]

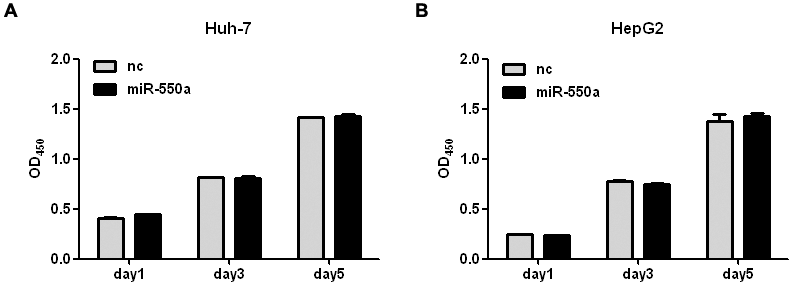

Supplement: Figure S2 — miR-550a has no significant effects on HCC cell growth in vitro. (A, B) CCK-8 assays of Huh-7 and HepG2 cells were performed every other day after transfection with a miR-550a mimic or negative control (nc). The data are presented as the mean ± S.E.M. (TIF) [file pone.0048958.s002.tif]

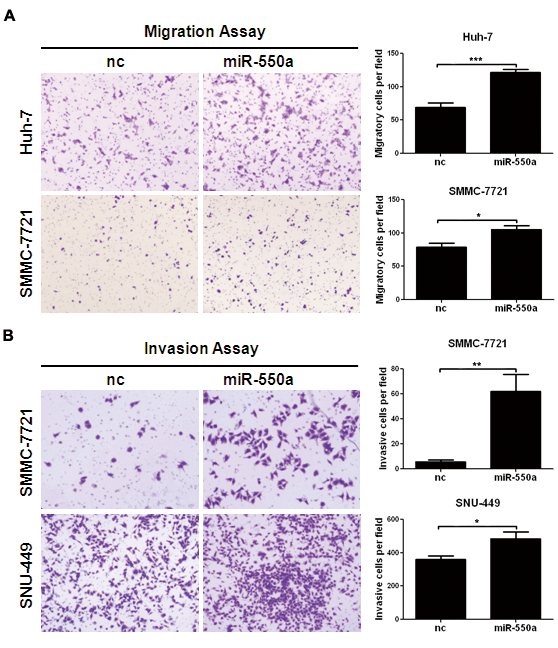

Supplement: Figure S3 — A miR-550a mimic facilitates HCC cell migration and invasion in vitro. (A) Transwell migration assays of Huh-7 and SMMC-7721 cells transfected with the miR-550a mimic or negative control (nc). (B) Transwell invasion assays of SMMC-7721 and SNU-449 cells transfected with the miR-550a mimic or nc. The values shown indicate the mean ± S.E.M. (TIF) [file pone.0048958.s003.tif]

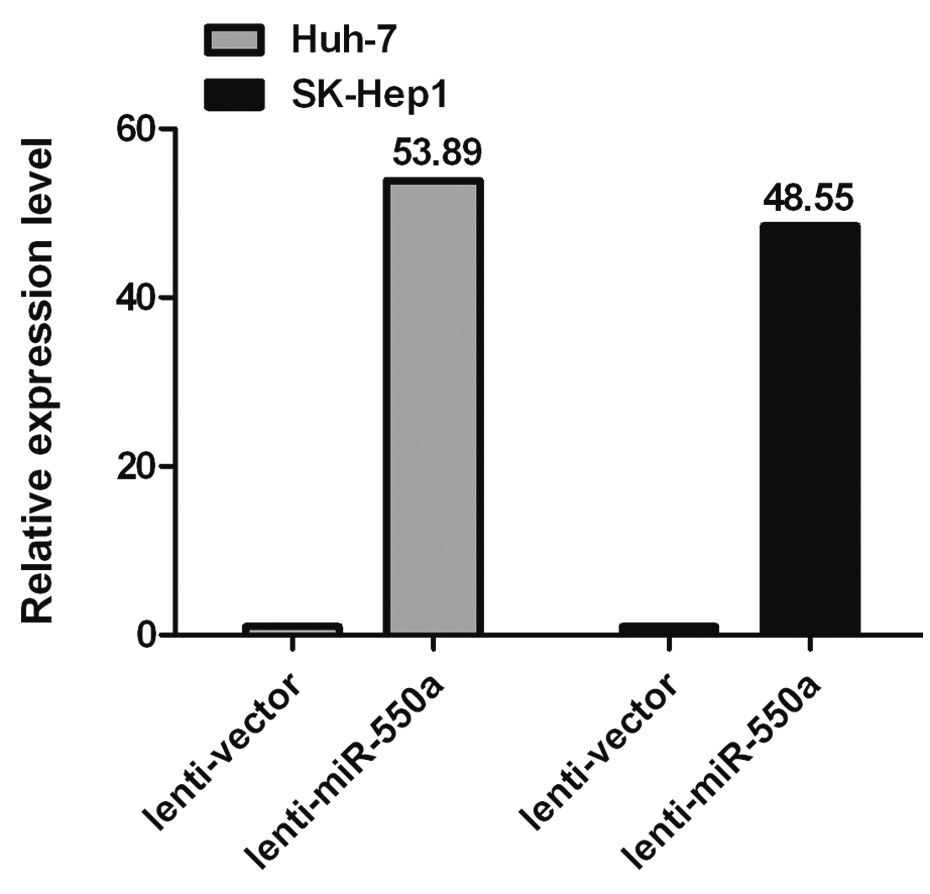

Supplement: Figure S4 — The expression of miR-550a in stable cell lines. The relative expression level of mature miR-550a was determined in Huh-7 and SK-Hep1 cells infected with pWPXL-miR-550a or control lentivirus. U6 snRNA was used as an internal control. (TIF) [file pone.0048958.s004.tif]

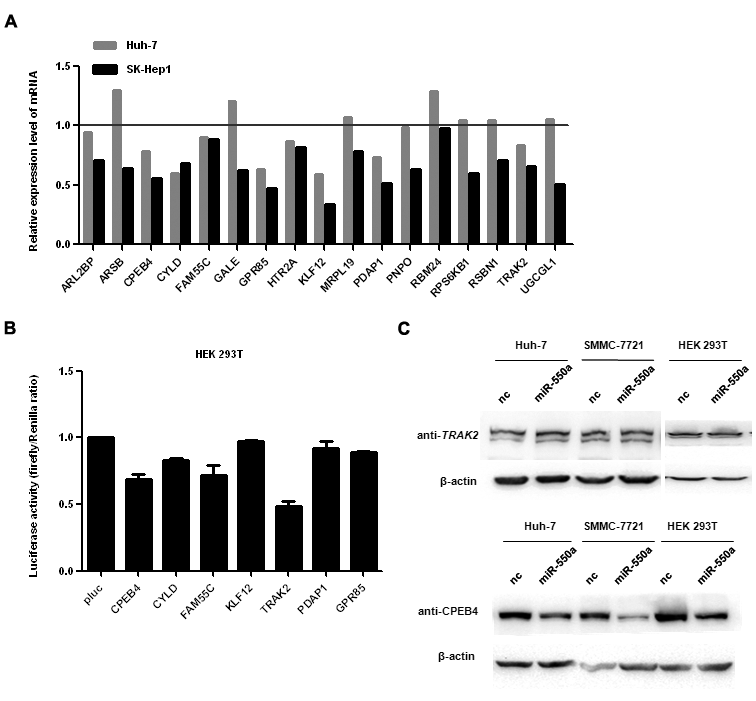

Supplement: Figure S5 — The identification of potential miR-550a target genes. (A) The mRNA expression levels of the predicted genes in Huh-7 and SK-Hep1 cells expressing miR-550a or vector were evaluated by real-time PCR. (B) Dual-luciferase activity assays were used to determine the binding potential between miR-550a and the 3′UTR of these candidate genes. Renilla luciferase activity was detected as an internal control. (C) Western blot assays of the TRAK2 and CPEB4 protein levels in Huh-7, SMMC-7721 and HEK 293T cells. (TIF) [file pone.0048958.s005.tif]
