# Supplementary material for: MicroRNA-550a Acts as a Pro-Metastatic Gene and Directly Targets Cytoplasmic Polyadenylation Element-Binding Protein 4 in Hepatocellular Carcinoma
Source: PLoS One. 2012 Nov 7;7(11):e48958. doi: 10.1371/journal.pone.0048958 (PMC3492136; doi:10.1371/journal.pone.0048958)
Supplement: Table S1 — The primer sequences for real-time PCR. (DOC) [file pone.0048958.s006.doc]

**Supporting Tables**

**Table S1 The primer sequences for real-time PCR**

| **Gene Name** | **Sequences** | |
| --- | --- | --- |
| *ARHGEF7* | Sense | 5'-TGATGACGCCTCTAAG-3' |
|  | Antisense | 5'-TGAAAGCGGATCTGTA-3' |
| *ARL2BP* | Sense | 5'-CTGGAGCGAGCCCTAT-3' |
|  | Antisense | 5'-TCCTACAGCCGAGACA-3' |
| *ARSB* | Sense | 5'-GCTGGCAGACGACCTA-3' |
|  | Antisense | 5'-TGTAAACCTGTACGGATC-3' |
| *CDIPT* | Sense | 5'-TTGCGGGTAGGAAGGG-3' |
|  | Antisense | 5'-GGGCACGAACAGGAAGAT-3' |
| *CPEB4* | Sense-1 | 5'-GCTACCAGAGTCCGTCAC-3' |
|  | Antisense-1 | 5'-CTTCAAAGGCGAGATG-3' |
|  | Sense-2 | 5'-CAGCCACTTGACCCAC-3' |
|  | Antisense-2 | 5'-AATCCCAGCGTAGCAC-3' |
| *CYLD* | Sense | 5'-GCAGAGTAGGGACGAG-3' |
|  | Antisense | 5'-GCAGAGGAGGAAACTAA-3' |
| *DAZAP2* | Sense | 5'-AAATGAATGTGGGTGAA-3' |
|  | Antisense | 5'-GCAAGGGATGGAAAGG-3' |
| *FAM55C* | Sense | 5'-TATCTACCGCACAAGC-3' |
|  | Antisense | 5'-TAAGGGCAGTCATCTC-3' |
| *GALE* | Sense | 5'-CATGCCTTATGTCTCCC-3' |
|  | Antisense | 5'-CGTGCCCAGGTTGTAG-3' |
| *GPR85* | Sense | 5'-TCCAACACCGCTCCT-3' |
|  | Antisense | 5'-TGACTGCTGCTACAAACT-3' |
| *HTR2A* | Sense | 5'-AGGTGCTGGGCATCGT-3' |
|  | Antisense | 5'-GTGGGTTGACTGCTGAAGA-3' |
| *KIAA1715* | Sense | 5'-GTGACTTGGCTACCTG-3' |
|  | Antisense | 5'-GCAACCCACTACCTAT-3' |
| *KLF12* | Sense | 5'-AAGCCATTCAAGTGCG-3' |
|  | Antisense | 5'-CGGGTAAAGACGGTTC-3' |
| *MRPL19* | Sense | 5'-GCCCTGGTCTAAACGC-3' |
|  | Antisense | 5'-GAGCCAATGTATCTTCTG-3' |
| *NHLH2* | Sense | 5'-CTCGCTACCTGTTCTC-3' |
|  | Antisense | 5'-CTTCTGCCCTCATTCT-3' |
| *PDAP1* | Sense | 5'-AAGTAACTGCGACCCG-3' |
|  | Antisense | 5'-GCTGTCCTGCATCTTTC-3' |
| *PNPO* | Sense | 5'-GAGGCTGTTCAGTGTCC-3' |
|  | Antisense | 5'-AGTGGCTCCCAGTAGA-3' |
| *RBM24* | Sense | 5'-CCAGGAGTAACAACGG-3' |
|  | Antisense | 5'-ACAGGACCTTCCGACT-3' |
| *RPS6KB1* | Sense | 5'-AGCATCCCTTCATCGT-3' |
|  | Antisense | 5'-CAGGCAGTGTCTTCCATA-3' |
| *RSBN1* | Sense | 5'-TGGGTCTGCTGCTACT-3' |
|  | Antisense | 5'-GAACCTGTGCCATAAA-3' |
| *TRAK2* | Sense | 5'-ACCAACCGACGAGATT-3' |
|  | Antisense | 5'-AAGGGCAAGGTGAGTG-3' |
| *UGCGL1* | Sense | 5'-ACCCAACCGCAGTGAA-3' |
|  | Antisense | 5'-TCCAAGGCAATGTCCC-3' |
