# Supplementary material for: MicroRNA-550a Acts as a Pro-Metastatic Gene and Directly Targets Cytoplasmic Polyadenylation Element-Binding Protein 4 in Hepatocellular Carcinoma
Source: PLoS One. 2012 Nov 7;7(11):e48958. doi: 10.1371/journal.pone.0048958 (PMC3492136; doi:10.1371/journal.pone.0048958)
Supplement: Table S2 — The nested PCR primer sequences for miR-550a or 3'UTR of potential target genes. (DOC) [file pone.0048958.s007.doc]

**Table S2 The nested PCR primer sequences for miR-550a or 3'UTR of potential target genes**

| **Gene Name** | **Sequences** | |
| --- | --- | --- |
| pre-miR-550a-F | Lateral | 5'-TGCTGTTAGGTTGTCTTCA-3' |
| pre-miR-550a-R |  | 5'-CTATGTTTTGTCCAATTTCT-3' |
| pre-miR-550a-F–*BamHⅠ* | Inside | 5’-cg ggatcc TGAGCCTTCTAACCACAAC-3' |
| pre-miR-550a-R–*EcoRⅠ* |  | 5'-cg gaattc TTCTCCTTACAAACCCAAA-3' |
| *CPEB4*-F | Lateral | 5'-CAGGCTTTAATGAACT-3' |
| *CPEB4*-R |  | 5'-TGGTAACTCCCAACTA-3' |
| *CPEB4*-*KpnⅠ* | Inside | 5'-GA GGTACC TGCTGAATTTCGGTAC-3' |
| *CPEB4*-*XhoⅠ* |  | 5'-GA CTCGAG ATTGTTTATGCCCTGT-3' |
| *CYLD*-F | Lateral | 5'-CTACCTGGAGGAATGA-3' |
| *CYLD*-R |  | 5'-CTAGGGATAGGAAGAAT-3' |
| *CYLD*-*KpnⅠ* | Inside | 5'-GA GGTACC TACTAAGGGCTGACGA-3' |
| *CYLD*-*XhoⅠ* |  | 5'-GA CTCGAG GCTGACAGTGAGGGAT-3' |
| *FAM55C*-F | Lateral | 5'-GGAAGGGACTGGAGGA-3' |
| *FAM55C*-R |  | 5'-ATGCCCAAATCAAGAA-3' |
| *FAM55C*-*KpnⅠ* | Inside | 5'-GA GGTACC ATCTTCTGGCGGTTAA-3' |
| *FAM55C*-*XhoⅠ* |  | 5'-GA CTCGAG CTGTCTACTTAGGAGCA-3' |
| *KLF12*-F | Lateral | 5'-GGCATAACTGCTTGAC-3' |
| *KLF12*-R |  | 5'-ATGGCAGAATGACAAG-3' |
| *KLF12*-*KpnⅠ* | Inside | 5'-GA GGTACC TGACGAGGCATTACCG-3' |
| *KLF12*-*XhoⅠ* |  | 5'-GA CTCGAG TTCATGCTGCTTGCTG-3' |
| *TRAK2*-F | Lateral | 5'-AGCCCAAATACCACTC-3' |
| *TRAK2*-R |  | 5'-CCATTCAGGGACACTT-3' |
| *TRAK2*-F-*KpnⅠ* | Inside | 5'-GA GGTACC TGGGTAGAGGGTGGGA-3' |
| *TRAK2*-R-*XhoⅠ* |  | 5'-GA CTCGAG GGGAACGAAAGGGAGA-3' |
| *PDAP1*-F | Lateral | 5'-AATAAGTAACTGCGACCCG-3' |
| *PDAP1*-R |  | 5'-CTCACTGCCACCTCTGC-3' |
| *PDAP1*-*KpnⅠ* | Inside | 5'-GA GGTACC TTTTCAGCCCTACCCAT-3' |
| *PDAP1*-*XbaⅠ* |  | 5'-GC TCTAGA GGCATACACCACCAAGC-3' |
| *GPR85*-F | Lateral | 5'-TGAACCACTGCCCTAA-3' |
| *GPR85*-R |  | 5'-TGTGCTTGCCTCCC-3' |
| *GPR85*-F-*KpnⅠ* | Inside | 5'-GA GGTACC GCCAAGGGAAATCA-3' |
| *GPR85*-R-*XhoⅠ* |  | 5'-GA CTCGAG AATTAACAGGCTACACG-3' |
| *HTR2A*-F | Lateral | 5'-TGCCAAGACAACAGATA-3' |
| *HTR2A*-R |  | 5'-GCCCAGGAGGAAAT-3' |
| *HTR2A*-F-*KpnⅠ* | Inside | 5'-GA GGTACC ACAATAGCGACGGAGTG-3' |
| *HTR2A*-R-*XhoⅠ* |  | 5'-GA CTCGAG CAGCCTTGAGCTTCTGG-3' |
| *CPEB4*-M-F |  | 5'-GAACATTTTTACCGTGACTTTTCACTGACGGGATA-3' |
| *CPEB4*-M-R |  | 5'-CAGTGAAAAGTCACGGTAAAAATGTTCACTGTTGG-3' |
