# Supplementary material for: MicroRNA-550a Acts as a Pro-Metastatic Gene and Directly Targets Cytoplasmic Polyadenylation Element-Binding Protein 4 in Hepatocellular Carcinoma
Source: PLoS One. 2012 Nov 7;7(11):e48958. doi: 10.1371/journal.pone.0048958 (PMC3492136; doi:10.1371/journal.pone.0048958)
Supplement: Table S3 — The information of miR-550a and its 3′UTR vectors. (DOC) [file pone.0048958.s008.doc]

**Table S3 The information of miR-550a and its 3’UTR vectors**

| **Gene Name** | **the length** | **the relative position of miR-550a / the miR550a binding sites** |
| --- | --- | --- |
| miR-550a vector | 466 | 247-343 |
| *CPEB4* | 831 | 113-119 |
| *CYLD* | 830 | 469-475 |
| *FAM55C* | 794 | 605-611 |
| *KLF12* | 610 | 259-265 |
| *TRAK2* | 1081 | 320-326，866-872 |
| *PDAP1* | 812 | 195-201 |
| *GPR85* | 479 | 275-281 |
