# Supplementary material for: MicroRNA-550a Acts as a Pro-Metastatic Gene and Directly Targets Cytoplasmic Polyadenylation Element-Binding Protein 4 in Hepatocellular Carcinoma
Source: PLoS One. 2012 Nov 7;7(11):e48958. doi: 10.1371/journal.pone.0048958 (PMC3492136; doi:10.1371/journal.pone.0048958)
Supplement: Table S4 — The sequences of siRNAs against CPEB4 . (DOC) [file pone.0048958.s009.doc]

**Table S4 The sequences of siRNAs against *CPEB4***

|  | **Sequences** | |
| --- | --- | --- |
| Si-*CPEB4*-1 | Sense | 5’-CUGCCUCAUUUGGCGAAUATT-3’ |
|  | Antisense | 5’-UAUUCGCCAAAUGAGGCAGTT-3’ |
| Si-*CPEB4*-2 | Sense | 5’-ccugcuguuucaagaugaatt-3’ |
|  | Antisense | 5’-UUCAUCUUGAAACAGCAGGTT-3’ |
| Si-*CPEB4*-3 | Sense | 5’-GCAGCAUGGAGAGAUAGAUTT-3’ |
|  | Antisense | 5’-AUCUAUCUCUCCAUGCUGCTT-3’ |
