# Supplementary material for: MicroRNA-550a Acts as a Pro-Metastatic Gene and Directly Targets Cytoplasmic Polyadenylation Element-Binding Protein 4 in Hepatocellular Carcinoma
Source: PLoS One. 2012 Nov 7;7(11):e48958. doi: 10.1371/journal.pone.0048958 (PMC3492136; doi:10.1371/journal.pone.0048958)
Supplement: Table S5 — The possible target genes for miR-550a in HCC cells. (DOC) [file pone.0048958.s010.doc]

**Table S5 The possible target genes for miR-550a in HCC cells**

| **Gene** | **Official Full Name** |
| --- | --- |
| *CPEB4* | Cytoplasmic polyadenylation element binding protein 4 |
| *NHLH2* | nescient helix loop helix 2 |
| *ARL2BP* | ADP-ribosylation factor-like 2 binding protein |
| *CDIPT* | CDP-diacylglycerol--inositol 3-phosphatidyltransferase |
| *KLF12* | Kruppel-like factor 12 |
| *ARHGEF7* | Rho guanine nucleotide exchange factor (GEF)7 |
| *RPS6KB1* | ribosomal protein S6 kinase, 70kDa, polypeptide 1 |
| *UGCGL1* | UDP-glucose glycoprotein glucosyltransferase 1 |
| *RSBN1* | round spermatid basic protein 1 |
| *HTR2A* | 5-hydroxytryptamine (serotonin) receptor 2A |
| *TRAK2* | trafficking protein, kinesin binding 2 |
| *GPR85* | protein-coupled receptor 85 |
| *KIAA1715* | KIAA1715 |
| *MRPL19* | mitochondrial ribosomal protein L19 |
| *GALE* | UDP-galactose-4-epimerase |
| *PDAP1* | PDGFA associated protein 1 |
| *DAZAP2* | DAZ associated protein 2 |
| *ARSB* | Arylsulfatase B |
| *LOC26010* | - |
| *FAM55C* | family with sequence similarity 55, member C |
| *RBM24* | RNA binding motif protein 24 |
| *CYLD* | cylindromatosis (turban tumor syndrome) |
| *PNPO* | pyridoxamine 5'-phosphate oxidase |
